# SCY-078 Is Fungicidal against Candida Species in Time-Kill Studies

**DOI:** 10.1128/AAC.01961-16

**Published:** 2017-02-23

**Authors:** Bernard Scorneaux, David Angulo, Katyna Borroto-Esoda, Mahmoud Ghannoum, Michael Peel, Stephen Wring

**Affiliations:** a Scynexis, Inc., Jersey City, New Jersey, USA; b Case Western University, Cleveland, Ohio, USA

**Keywords:** SCY-078

## Abstract

SCY-078 is an orally bioavailable ß-1,3-glucan synthesis inhibitor (GSI) and the first-in-class of structurally novel triterpene antifungals in clinical development for treating candidemia and invasive candidiasis. *In vitro* susceptibilities by broth microdilution, antifungal carryover, and time-kill dynamics were determined for three reference (ATCC) strains (Candida albicans 90028, Candida parapsilosis 90018, and Candida tropicalis 750), a quality-control (QC) strain (Candida krusei 6258), and four other strains (C. albicans MYA-2732, 64124, and 76485 and Candida glabrata 90030). Caspofungin (CASP), fluconazole (FLC), and voriconazole (VRC) were comparators. For time-kill experiments, SCY-078 and CASP were evaluated at 0.25, 1, 2, 4, 8, and 16 times the MIC_80_, and FLU and VRC were evaluated at 4× MIC_80_. The time to reach 50%, 90%, and 99.9% reduction in the number of CFUs from the starting inoculum was determined. Net change in the number of CFU per milliliter was used to determine 50% and 90% effective concentrations and maximum effect (EC_50_, EC_90_, and *E*_max_, respectively). The SCY-078 MIC range was between 0.0625 and 1 μg/ml and generally similar to that of CASP. Antifungal carryover was not observed for SCY-078. SCY-078 was fungicidal against seven isolates at ≥4× MIC (kill of ≥3 log_10_) and achieved a 1.7-log_10_ reduction in CFU count/milliliter against C. albicans 90028. CASP behaved similarly against each isolate and achieved a 1.5-log_10_ reduction in the number of CFU/milliliter against C. albicans 90028. Reductions of 50% in CFU count/milliliter were achieved rapidly (1 to 2.8 h); fungicidal endpoints were reached at 12.1 to 21.8 h at ≥4× MIC. EC_90_ was reached at ∼5× MIC at each time point to 24 h. The EC_50_ and EC_90_ values were generally similar (8 to 24 h). Time-kill behavior of CASP was similar to that of SCY-078. FLC and VRC were fungistatic. Overall, SCY-078 has primarily fungicidal activity against Candida spp. and behaved comparably to CASP.

## INTRODUCTION

Candida species remain the most common cause of invasive fungal infections, with disseminated candidiasis ranked as the fourth most common cause of nosocomial bloodstream infections in the Unites States (1–3). Because candidiasis tends to occur in the sickest of patients, it is associated with approximately 40% mortality (4). Treatment options for candidiasis are generally restricted to antifungals belonging to the polyene, triazole, and echinocandin classes of drugs. Although these agents demonstrate high levels of antifungal activity, their use can be hampered by toxicity, poor tolerability, or a narrow activity spectrum. Polyenes (e.g., amphotericin B) are associated with high rates of toxicity and side effects, and drug-drug interactions are a major disadvantage of the azoles (1, 4). The echinocandins have a lower incidence of adverse events than other antifungals; however, they are only available as intravenous (i.v.) formulations (5). Importantly, the recent emergence of antifungal drug resistance is worrying, and in some settings the evolution of multidrug-resistant strains insensitive to both azoles and echinocandins is a major concern (6). Candida resistance to echinocandins has emerged over recent years and is most commonly associated with Candida glabrata, with resistance rates of >10% at selected institutions (7). Drug resistance is a major concern recognized by the Centers for Disease Control and Prevention (CDC), with Candida infections now associated with a “serious” hazard level, indicating that these infections have a significant threat of resistance. The CDC further predicts that these infections pose a threat that will worsen and may become urgent (8). Taken together, the side effects of existing antifungal drugs along with the emergence of drug-resistant organisms highlight the need for the development of novel antifungal compounds that are more effective and safe and target resistant fungi.

To address this unmet need, SCY-078 is being developed as a novel, intravenous and orally bioavailable antifungal. Earlier studies showed that SCY-078 has broad, potent activity against Candida in vitro (9, 10) and possesses efficacy in murine animal models of invasive candidiasis (11). SCY-078 inhibits fungal glucan synthesis (GS); however, it is structurally distinct from currently available glucan synthase inhibitors (echinocandins) (12).

Additionally, SCY-078 retains *in vitro* activity against both azole-resistant and the majority of echinocandin-resistant strains of Candida species (9, 10), indicating that its mechanism of action is not markedly affected by key resistance mechanisms or mutations associated with traditional targets.

Echinocandins and amphotericin B have demonstrated *in vitro* fungicidal activity, and recent reviews of published clinical data suggest that treatment with fungicidal agents, particularly early in therapy, may result in clinical benefit, especially in neutropenic patients (1, 13).

In this study, we characterized the concentration- and time-dependent relationships between SCY-078 and the rate, extent, and cidality of its antifungal activity against isolates of several Candida spp. In addition, we compared the antifungal time-kill kinetics and *in vitro* activity of SCY-078 to selected approved azoles (fluconazole [FLC] or voriconazole [VRC]) and a representative echinocandin (caspofungin [CASP]).

## RESULTS

### Antifungal susceptibility.

*In vitro* susceptibilities are summarized for three Candida reference strains (C. albicans 90028, C. parapsilosis 90018, and C. tropicalis 750) and five other isolates of Candida spp. to SCY-078, caspofungin, fluconazole, and voriconazole (Table 1). The MICs were determined after 24 h of incubation with three different methods of endpoint determination: visual with and without shaking and spectrophotometric readings.

**TABLE 1:** Comparison of broth microdilution MICs read by the visual and spectrophotometric methods for seven Candida strains^*a*^

Strain	Antifungal agent	Reference range (μg/ml)^*b*^	Modal MIC (μg/ml) by inspection method^*c*^		
			V	VS	SP
C. albicans ATCC 90028	FLC	0.25–1.0	0.25	0.25	0.25
	VRC	NA^d^	0.0625	0.0625	0.0625
	SCY-078	NA	0.0625	0.0625	0.0625
	CASP	NA	0.5	0.5	0.125
C. albicans ATCC MYA2732	FLC	NA	>16	>16	>16
	VRC	NA	0.125	0.125	0.25
	SCY-078	NA	0.0625	0.0625	0.0625
	CASP	NA	0.125	0.125	0.125
C. albicans ATCC 64124	FLC	NA	>16	>16	>16
	VRC	NA	>16	>16	>16
	SCY-078	NA	0.25	0.25	0.25
	CASP	NA	0.0625	0.0625	0.0625
C. albicans ATCC 76485	FLC	NA	0.5	0.5	0.5
	VRC	NA	0.0625	0.0625	0.0625
	SCY-078	NA	0.5	0.5	0.5
	CASP	NA	0.125	0.125	0.125
C. krusei ATCC 6258	FLC	16–64	>16	>16	>16
	VRC	0.06–0.5	0.25	0.25	0.25
	SCY-078	NA	1	1	1
	CASP	0.125–1	0.5	0.5	0.5
C. glabrata ATCC 90030	FLC	NA	>16	>16	>16
	VRC	NA	0.25	0.25	0.25
	SCY-078	NA	1	1	1
	CASP	NA	0.5	0.5	0.5
C. parapsilosis ATCC 90018	FLC	0.25–1	1	1	1
	VRC	NA	0.125	0.125	0.125
	SCY-078	NA	0.125	0.125	0.125
	CASP	NA	0.0625	0.0625	0.0625
C. tropicalis ATCC 750	FLC	1–4	1	1	1
	VRC	NA	0.125	0.125	0.125
	SCY-078	NA	0.125	0.125	0.125
	CASP	NA	0.0625	0.0625	0.0625

a All isolates were tested nine times over 3 days according to the microdilution method of CLSI document M27-A3 (14).

b Reference ranges were established by the broth macrodilution or microdilution method (CLSI document M27-S4 [15]). NA, not available.

c MICs were determined after 24 h of incubation; endpoints were measured at 492 nm. V, visual inspection; VS, visual inspection with agitation; SP, spectrophotometric inspection. Spectrophotometric endpoints were determined as concentrations resulting in a ≥50% inhibition of growth.

Agreement among the three methods of reading did not differ by more than 2 doubling dilutions. In each experiment, the visual MICs at 24 h for the reference strains and the quality-control (QC) strain C. krusei 6258 were within the accepted limits as established by CLSI documents M27-A3 and M27-S4 (14, 15). SCY-078 MICs ranged from 0.0625 to 1 μg/ml, consistent with values obtained from earlier studies (16–18). MICs against C. glabrata and C. krusei isolates were higher than those for other species. SCY-078 MIC values were generally comparable to those of caspofungin against the strains tested in this study.

### Limit of quantitation.

Using a 30-μl sampling volume, the lower limit of accurate and reproducible quantitation was 30 CFU/ml for each of the isolates. According to this sampling method, the cumulative (all species) percent coefficient of variation (% CV) of CFU counts resulting from control samples was 22.7%. The presence of antifungals in solution did not affect the reproducibility of sampling results.

### Antifungal carryover.

Antifungal carryover was not observed at any of the SCY-078 or caspofungin concentrations for any of the isolates tested (data not shown).

### Killing curves.

**FIG 1:**
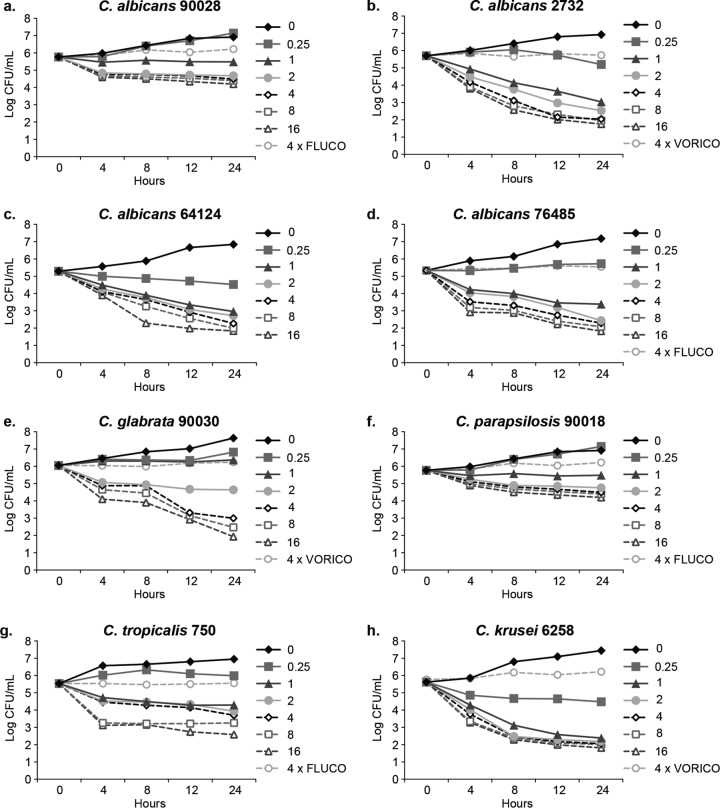
Time-kill curve plots for the indicated Candida species at the indicated SCY-078 MIC_80_ multiples. ◆, control (0× MIC). FLUCO, fluconazole; VORICO, voriconazole.

**FIG 2:**
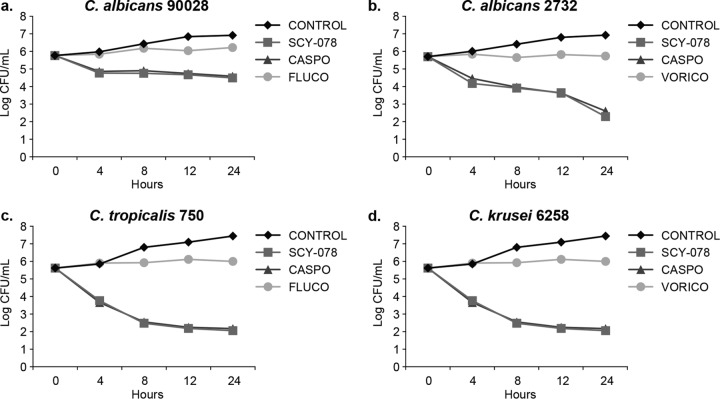
Time-kill curves for SCY-078, caspofungin (CASPO), fluconazole (FLUCO), and voriconazole (VORICO) at 4 times the MIC_80_ against the indicated Candida species and a control. 2732, MYA-2732.

A time-kill plot (Fig. 1) of the activity of SCY-078 against each Candida species tested revealed that SCY-078 exhibited concentration- and time-dependent fungicidal activity (defined as a kill of ≥log 3) against seven (C. albicans MYA-2732, 64124, and 76485 strains, C. krusei, C. glabrata, C. parapsilosis, and C. tropicalis) of the eight isolates tested. A median value of 4× MIC (range, 1× to 16×MIC) was associated with achieving the cidal endpoint at 24 h across the seven isolates. The equivalent median concentration was 2 μg/ml (range, 0.25 to 4 μg/ml). SCY-078 exhibited fungistatic activity (in so much as it did not achieve a ≥3-log reduction in CFU count/milliliter) against the remaining strain (C. albicans 90028); nonetheless, the CFU count/milliliter was reduced by 1.5-log_10_ CFU/ml. In contrast, there was no measurable decrease in CFU count/milliliter for the FLC comparator, which demonstrated only static behavior (described below). CASP displayed comparable activity to SCY-078 against this strain (Fig. 2). CASP showed cidal behavior against six of the isolates tested since it narrowly missed achieving the cidality endpoint for C. tropicalis (2.8 log_10_ CFU/ml versus 3.0 log_10_ CFU/ml for SCY-078). A median value of 10× MIC (range, 1× to 16× MIC) was associated with achieving the cidal endpoint at 24 h across the six isolates. The equivalent median concentration was 1 μg/ml (range, 0.5 to 2 μg/ml). In contrast to SCY-078 and CASP, the azole comparators (FLC and VRC) when tested at 4 times their MICs demonstrated no measurable decrease in CFU count/milliliter, reflecting the anticipated fungistatic behavior (Table 1).

For SCY-078 the times to reach 50%, 90%, and 99.9% reductions in the number of CFU from the starting inoculum with each multiple of the SCY-078 MIC for each isolate are summarized in Table 2. A 50% reduction was rapidly achieved between 1 and 2.8 h for all isolates. The time required to reach a 90% reduction in the number of CFU/milliliter ranged between 3.4 and 15.5 h. Similar times to achieve 50%, 90%, and 99.9% reductions in the number of CFU/milliliter across the isolates indicated that SCY-078 had comparable activity against these organisms. Caspofungin showed times comparable to those of SCY-078 for each isolate to reach 50%, 90%, and 99.9% reductions in the number of CFU (data not shown).

**TABLE 2:** Times for SCY-078 to achieve 50%, 90%, and 99.9% reductions in growth from starting inoculum

Strain and growth reduction	Median time (h) at the following MIC_80_ multiple^*a*^					
	0.25	1	2	4	8	16
C. albicans ATCC 90028						
50%	NR	NR	2.3	2.2	2.2	2.1
90%	NR	NR	11.5	9.2	8.8	7.5
99.9%	NR	NR	NR	NR	NR	NR
C. albicans ATCC MYA2732						
50%	NR	1.7	1.9	1.9	1.3	1.2
90%	NR	7.6	6.4	6.3	4.2	4.0
99.9%	NR	NR	NR	19.9	13.5	12.5
C. albicans ATCC 64124						
50%	NR	1.9	1.7	1.6	1.4	1.1
90%	NR	6.3	5.6	5.5	4.8	3.7
99.9%	NR	NR	NR	21.8	16.2	12.1
C. albicans ATCC 76485						
50%	NR	1.6	1.8	1.5	1.3	1.3
90%	NR	5.4	6.0	5.00	4.3	4.2
99.9%	NR	NR	NR	19.4	14.8	13.6
C. krusei ATCC 6258						
50%	1.3	1.2	1.1	1.1	1.0	1.0
90%	5.7	4.1	3.6	3.5	3.4	3.5
99.9%	NR	14.1	11.7	11.4	10.7	10.9
C. glabrata ATCC 90030						
50%	NR	NR	1.7	1.4	1.4	1.3
90%	NR	NR	6.3	4.6	4.7	4.4
99.9%	NR	NR	NR	18.2	14.8	13.5
C. parapsilosis ATCC 90018						
50%	NR	2.8	1.9	2.0	1.9	1.4
90%	NR	15.5	7.8	6.8	6.3	4.7
99.9%	NR	NR	NR	NR	NR	16.6
C. tropicalis ATCC 750						
50%	NR	2.7	1.9	2.4	1.3	1.3
90%	NR	9.6	8.8	6.3	4.4	4.3
99.9%	NR	NR	NR	NR	NR	>24.0

a MIC_80_ values were determined as a more stringent criterion than MIC_50_ (19). NR, not reached.

SCY-078 exhibited fungicidal activity (99.9% reduction) after 12.1 to 21.8 h at ≥4 times the MIC against C. albicans MYA-2732, 64124, and 76485. For the non-albicans species, SCY-078 achieved cidality against C. glabrata 90030 at ≥4 times the MIC and reached the fungicidal endpoint after 13.5 to 18.2 h. SCY-078 also showed fungicidal activity against C. krusei 6258 beginning at 1 times the MIC, and the fungicidal endpoint was attained between 10.7 to 14.1 h. SCY-078 at 16 times the MIC demonstrated fungicidal activity against C. parapsilosis 90018 and C. tropicalis 750 after 16.6 h and at 24 h, respectively.

**FIG 3:**
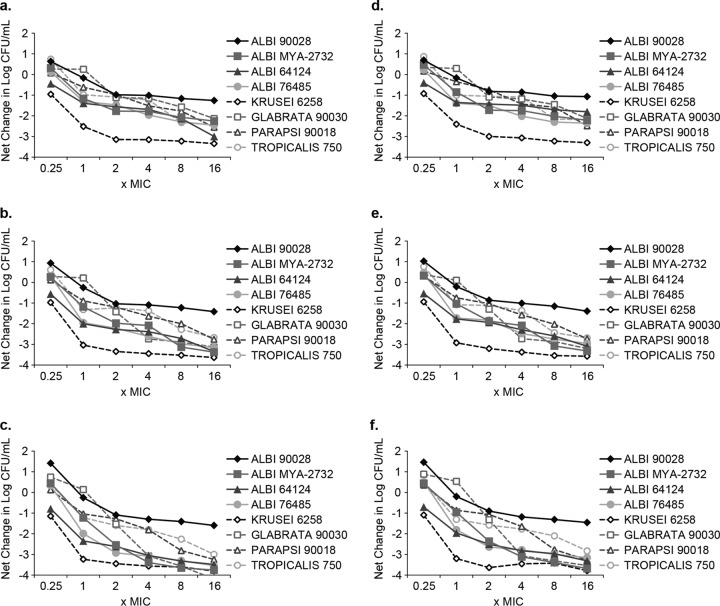
Composite concentration-response curves for SCY-078 (a to c) and caspofungin (d to e) versus all test isolates following exposure for 8 h, 12 h, and 24 h. Albi, C. albicans; Krusei, C. krusei; Glabrata, C. glabrata; Parapsi, C. parapsilosis; Tropicalis, C. tropicalis.

The antifungal activity assessed as net change in CFU count/milliliter following 8, 12, and 24 h of exposure of the eight isolates to SCY-078 or CASP was plotted as a function of the multiple of the MIC (Fig. 3). Antifungal activity typically increased with concentration and incubation time, with maximal responses (maximum effect, *E*_max_) generally occurring at the highest drug concentrations following 24-h incubations. These results indicate that SCY-078 produces fungicidal activity based on concentration and time of exposure, consistent with the pharmacokinetic/pharmacodynamic (PK/PD) parameter of the area under the concentration-time curve (AUC)/MIC.

Composite 50% and 90% effective concentrations (EC_50_ and EC_90_, respectively) and *E*_max_ results are summarized for SCY-078 and CASP in Table 3. Values for EC_50_ and EC_90_ are expressed relative to the maximal reduction in CFU count/milliliter at each incubation time point. The concentrations that produced either 50% or 90% of the *E*_max_ (EC_50_ or EC_90_, respectively) at each time point were approximately 1.5 or 5 times the MIC. This value is similar to the values determined for CASP (Table 3) and to those reported for micafungin (16).

**TABLE 3:** Composite *E*_max_ model parameters for SCY-078 and caspofungin

Time (h)	SCY-078			Caspofungin		
	MIC multiple for:		*E*_max_ (log_10_ CFU/ml)^*a*^	MIC multiple for:		*E*_max_ (log_10_ CFU/ml)
	EC_50_	EC_90_		EC_50_	EC_90_	
8	1.6	5.6	−2.4 ± 0.62	1.4	4.7	−2.2 ± 0.63
12	1.5	5.3	−2.9 ± 0.69	1.7	6.1	−2.9 ± 0.67
24	1.4	4.8	−3.3 ± 0.79	1.4	4.3	−3.1 ± 0.74

a *E*_max_, maximal reduction in log_10_ CFU/ml.

## DISCUSSION

In this study, we used four well-characterized strains (reference and QC) of Candida spp. to establish the reproducibility of the visual (with and without agitation) and spectrophotometric microdilution endpoint methods in the determination of the MIC and their levels of agreement with the M27-A3 and M27-S4 guidelines. These endpoints from 24-h incubations were reproducible and in agreement with one another. Extension of these studies to include four more Candida species demonstrated an excellent performance of all three methods for testing five different antifungal agents. The extent of activity afforded by SCY-078 did not appear to be dependent on the susceptibility of the isolate to fluconazole, as was evident by SCY-078's similar activity against fluconazole-susceptible and -resistant isolates of C. albicans. SCY-078 exerted antifungal effects against diverse Candida species and was at least as effective as caspofungin.

Time-kill curve studies demonstrated that SCY-078 killed eight Candida isolates at concentrations above the MIC, achieving fungicidal levels against seven isolates. The time to achieve 50%, 90%, and 99.9% reductions in growth from the starting inocula suggested a trend to shorter times with increasing concentrations. The kill curves obtained with SCY-078 indicate that the drug is primarily fungicidal (>99.9% reductions in the number of CFU per milliliter) against the C. albicans and non-albicans isolates tested. SCY-078, like CASP, was fungistatic (<99.9% reduction in growth) for C. albicans 90028 although the CFU count/milliliter was reduced by 1.5 log_10_ CFU/ml. Previous studies have shown that caspofungin, anidulafungin, and micafungin also exhibit predominantly fungicidal activity against Candida species, but fungistatic activity may also be observed, depending on the isolate and test conditions (16–18), in line with the results for SCY-078 and CASP in this study. In assessing our results, we found that concurrent time-kill experiments on isolates with fluconazole or voriconazole were consistent with growth inhibition but failed to show reductions in starting inocula, indicating only stasis and confirming previous observations (19). The fungicidal activity of SCY-078 against the non-albicans species is important given the acquired and intrinsic resistance to fluconazole (according to the CDC [http://www.cdc.gov/fungal/antifungal-resistance.html]).

When the pharmacodynamic characteristics of an antifungal drug are evaluated, it is important to determine the effect of concentration on both the rate and extent of *in vitro* activity. In the case of SCY-078, the time to reach a 50% or 90% reduction in growth rate occurred over the range 1.1 to 2.8 or 3.7 to 15.5 times the MIC, respectively, and the time to attain these endpoints trended shorter with increasing concentrations. These data are consistent with an agent exhibiting concentration- and time-dependent activity. This behavior is supportive of the AUC/MIC being a PK/PD parameter driving efficacy *in vivo*. The concentration-effect relationships observed with SCY-078 are comparable to those observed in this study for caspofungin and are generally similar to those reported previously for micafungin against 10 isolates of Candida species (16). The pharmacokinetic/-dynamic endpoints (AUC/MIC) associated with efficacy in murine models of disseminated candidiasis have been reported previously (11, 20).

Our data show that MIC values of SCY-078 using three endpoint microdilution methods were in agreement and that SCY-078 possesses potent and rapid *in vitro* activity against Candida that is primarily fungicidal against both azole-susceptible and -resistant isolates, with exposure concentration and time being key determinants of activity.

## MATERIALS AND METHODS

### Fungal isolates.

Four Candida albicans strains (ATCC 90028, ATCC MYA-2732, ATCC 64124, and ATCC 76485) and one strain each of Candida krusei (strain ATCC 6258), Candida glabrata (strain ATCC 90030), Candida parapsilosis (strain ATCC 90018), and Candida tropicalis (strain ATCC 750) were used in this study. All isolates were wild type (WT) with resistance to *fks* mutations. All the isolates were obtained from the American Type Culture Collection (ATCC, Manassas, VA).

### Antifungal agents.

The following antifungals were used in this study: SCY-078 (Scynexis, Inc., Jersey City, NJ), caspofungin (CASP; Lanospharma Laboratories Co., Ltd., Hong Kong), voriconazole (VRC; Sigma-Aldrich), and fluconazole (FLC; Sigma-Aldrich). Stock solutions (10 mg/ml) of these antifungals were prepared in dimethyl sulfoxide (DMSO; Sigma-Aldrich). Working solutions were prepared in RPMI 1640 medium (Sigma-Aldrich) buffered to a pH of 7.0 with 0.165 M morpholine-propanesulfonic acid (MOPS; Sigma-Aldrich). The final concentration of DMSO was ≤1% (vol/vol) of the solution composition. Stock solutions were separated into unit-of-use portions and stored at −80°C until used.

### Inoculum preparation.

A single colony of each isolate to be tested was grown overnight on Sabouraud dextrose agar (SDA) at 35°C and was then subcultured on the same medium for a further 24 h at the same temperature. The inoculum was prepared by diluting colonies of the overnight culture with sterile 0.9% NaCl. The resulting suspension was then vortexed for 15 s, and the cell density was adjusted spectrophotometrically with sterile saline to increase the transmittance to that produced by a 0.5 McFarland standard (barium sulfate; BD BBL, Sparks, MD) at a 530-nm wavelength. This suspension was then diluted to obtain an inoculum of 1 × 10^3^ to 5 × 10^3^ CFU/ml. Inoculum size was verified by plating 30 μl of serial dilutions of each inoculum onto an SDA plate, with incubation until colony growth became visible.

### Antifungal susceptibility.

The MICs of the tested antifungals for each test isolate were determined using broth microdilution techniques recommended by the Clinical and Laboratory Standards Institute (14) using both visual inspection (VI) and spectrophotometry. Briefly, an inoculum of 1 × 10^3^ to 5 × 10^3^ CFU/ml in RPMI 1640 medium buffered to a pH of 7.0 with MOPS was added (100 μl) to each well of microtiter trays containing 100 μl of antifungal drug solution. Microtiter trays were incubated at 35°C in a moist, dark chamber, and MICs were recorded after 24 h of incubation.

Visual inspection of the endpoint was determined with and without agitation. The broth microdilution wells that had not been agitated were read visually with the aid of a reading mirror; the growth in each well was compared with that in the growth control (drug-free) well. A numerical score, which ranged from 0 to 4, was given to each well according to the following scale recommended by CLSI: 0, optically clear; 1, slightly hazy; 2, prominent decrease in turbidity; 3, slight reduction in turbidity; and 4, no reduction in turbidity. The MIC values were defined as the lowest concentrations at which scores of 2 (prominent decrease in turbidity) were observed. Experiments were conducted in quintuplicate. Endpoint determinations were performed by means of visual and spectrophotometric determinations (21).

Visual inspection of the MIC endpoint with agitation was accomplished by first sealing the tops of the trays with clear tape (Titertek plate sealer tape; Flow Laboratories), placing the microtiter plate in a SpectraMax plate reader (Molecular Devices, San Diego, CA), and then mixing the samples (approximately 25 s) until a homogeneous yeast suspension was obtained in each well. The MIC endpoints for the agitated trays were defined as described above for the reference MICs.

Spectrophotometric endpoint readings of each well were performed with a SpectraMax plate reader (Molecular Devices, San Diego, CA) set at 492 nm after the wells had been agitated for 20 s. The percentage of growth in each well was calculated as the optical density (OD) of each well/OD of the drug-free well after subtraction of the background OD obtained from microorganism-free microtiter plates processed in the same manner as the inoculated plates. The spectrophotometric MIC endpoints were determined as the first concentration of the antifungal agent at which turbidity in the well was ≥80% less (MIC_80_ for SCY-078, caspofungin, fluconazole, and voriconazole) than that in the control well. Each experiment was performed in duplicate on seven different dates, and the results were reported as modal values. The 80% reduction was selected to provide a more stringent endpoint (19).

### Limit of quantitation.

The lower limit of accurately detectable CFU count/milliliter or the limit of quantitation was determined for each of the tested isolates. A fungal suspension was made in sterile water with each isolate and adjusted to a 0.5 McFarland turbidity standard (approximately 1 × 10^6^ to 5 × 10^6^ CFU/ml). A series of dilutions using sterile water were made, with the standardized suspensions resulting in three suspensions with fungal concentrations of approximately 100, 50, and 30 CFU/ml for each isolate. Thirty microliters was removed from each suspension and plated on SDA plates for CFU count determination. Plates were incubated at 35°C, and CFU counts were determined after 24 h and 48 h. Experiments were conducted in triplicate.

### Antifungal carryover.

The extent of antifungal carryover was evaluated before the time-kill curve studies were performed. Briefly, a fungal suspension was prepared with each test isolate to yield an inoculum of approximately 5 × 10^3^ CFU/ml. One hundred microliters of these suspensions was added to 900 μl of sterile water or sterile water plus antifungal drugs, resulting in a starting inoculum of approximately 5 × 10^2^ CFU/ml. Antifungal carryover was evaluated over a range of antifungal concentrations (from 0.25 to 16 times the MIC). Immediately following addition of the fungal inoculum to the aqueous solutions, 30-μl aliquots were removed and plated without dilution on SDA plates for determination of CFU counts. Following 48 h of incubation at 35°C, the number of CFU was counted. The mean CFU count for each antifungal, at each multiple of the MIC tested, was compared with that of the control. Significant antifungal carryover was defined as a reduction in the mean number of CFU/milliliter of >25% compared with value of the growth control. Tests were conducted in triplicate.

### Time-kill curves.

Time-kill experiments were conducted with RPMI 1640 medium buffered with MOPS as the growth medium. SCY-078 and caspofungin were tested over a range of concentrations: 0 (control), 0.25, 1, 2, 4, 8, and 16 times the MIC for each test isolate. Fluconazole and voriconazole were tested at 4 times the MIC for each test isolate. Prior to time-kill evaluation, isolates were subcultured twice on SDA plates. Colonies from fungal cultures, following incubation for 24 to 48 h, were suspended in 9 ml of sterile water and adjusted to a 0.5 McFarland turbidity standard. One milliliter of the adjusted fungal suspension was then added to growth medium alone (control) or to a solution of RPMI medium plus an appropriate amount of antifungal drug stock solution. These procedures resulted in a starting inoculum of approximately 1 × 10^5^ to 5 × 10^5^ CFU/ml, confirmed by the spectrophotometric method. Test solutions were placed on an orbital shaker and incubated with agitation at 35°C. At predetermined time points, 100-μl aliquots were obtained from each solution and serially diluted in sterile water, and 30 μl was plated on SDA plates for determination of CFU counts. Colony counts were determined following incubation at 35°C for 24 to 48 h. All time-kill experiments were performed in duplicate.

### Analysis.

Mean CFU counts (log_10_ CFU/milliliter) were plotted as a function of time for each isolate at each concentration of antifungal tested. Time-kill data were characterized as fungicidal or fungistatic as follows: fungicidal activity was defined as a ≥3-log_10_ (99.9%) reduction in numbers of CFU from the starting inoculum, and fungistatic activity was defined as a <99.9% reduction in growth from the starting inoculum. The time-kill data were fitted by using a single three-parameter exponential decay model (*y* = *y*_0_ + *ae*^−*bx*^) using SigmaPlot for Windows (version 12.0; SPSS, Inc.) to determine the time (in hours) required to reach 50%, 90%, and 99.9% reductions in growth from the starting inoculum as a measure of the rate of activity. The net change (log_10_ CFU/milliliter) in fungal density at each time point was determined for each isolate at each multiple of the MIC that inhibited 50% of isolates (MIC_50_) and plotted. This plot was fitted to a three-parameter sigmoidal Hill model [*y* = (*ax*^*b*^)/(*c*^*b*^ + *x*^*b*^)] (SigmaPlot) to determine the concentrations producing 50% of the maximal effect (EC_50_), 90% of the maximal effect (EC_90_), and the maximal effect (*E*_max_) to quantify the extent of activity. Figures 1 to 3 were prepared using GraphPad Prism, version 6.
